# Comparative study of the around-Fermi electronic structure of 5*d* metals and metal-oxides by means of high-resolution X-ray emission and absorption spectroscopies

**DOI:** 10.1107/S1600577520003690

**Published:** 2020-04-14

**Authors:** Anna Wach, Jacinto Sá, Jakub Szlachetko

**Affiliations:** a Institute of Nuclear Physics, Polish Academy of Sciences, PL-31342 Krakow, Poland; b Physical Chemistry Division, Department of Chemistry, Ångström Laboratory, Uppsala University, 75120 Uppsala, Sweden; c Institute of Physical Chemistry, Polish Academy of Sciences, 01-224 Warsaw, Poland

**Keywords:** X-ray absorption spectroscopy, X-ray emission spectroscopy, electronic structures, crystal field splitting, tungsten

## Abstract

The composition of occupied and unoccupied electronic states in the vicinity of Fermi energies is vital for all materials and relates to their physical, chemical and mechanical properties. This work demonstrates how the combination of resonant and non-resonant X-ray emission spectroscopies supplemented with theoretical modelling allows for quantitative analysis of electronic states in 5*d* transition metal and metal-oxide materials. Application of X-rays provides element selectivity that, in combination with the penetrating properties of hard X-rays, allows determination of the composition of electronic states under working conditions, *i.e.* non-vacuum environment. Tungsten metal and tungsten oxide are evaluated to show the capability to simultaneously assess composition of around-band-gap electronic states as well as the character and magnitude of the crystal field splitting.

## Abstract 2

This work demonstrates how the combination of resonant and non-resonant X-ray emission spectroscopies supplemented with theoretical modelling allows for quantitative analysis of electronic states around Fermi, band gap energies and strength of crystal field splitting in 5*d* transition metal and metal-oxide materials.

## 1. Introduction   

Compounds based on transition metals constitute one of the most interesting classes of materials with a variety of compositions and geometrical structures (Chen *et al.*, 2019 ▸; van Gog *et al.*, 2019 ▸; Guo *et al.*, 2015 ▸). They exhibit unique properties, such as superconductivity, magnetoresistance, ferroelectricity, charge ordering, thermoelectricity, and optical and catalytic activity, which originate from the outer *d*-valence electrons of transition metal elements. This makes them attractive materials in areas such as optics, electronics, catalysis, sensors, and energy conversion and storage (Kumaravel *et al.*, 2019 ▸; Najafi-Ashtiani *et al.*, 2019 ▸; Wang *et al.*, 2018*a* ▸). The functional properties of transition-metal-based materials are very sensitive to the electronic band structure of the central transition metal and are thereby strongly influenced by the elemental composition and the presence of defects or lattice distortions (Yu *et al.*, 2018 ▸). Thus, knowledge about the electronic structure of transition metal elements, especially orbital contribution and the relative energy position of the highest occupied (valence band) and the lowest unoccupied states (conduction band) is crucial for the design of innovative and efficient metal-oxide materials.

Current research efforts are focused on the discovery of more efficient materials with improved properties for cutting-edge applications. Conventional trial-and-error approaches, *i.e.* synthesis of a large number of materials, followed by screening of their properties, is unfortunately time- and resource-consuming. Recently, we proposed an innovative and cost-effective strategy for the rational design of oxynitride materials based on 3*d* transition metals, namely titanium (Sá *et al.*, 2018 ▸; Szlachetko & Sá, 2013 ▸). This approach is based on a combination of resonant X-ray emission spectroscopy (RXES) experiments and theoretical calculations to determine the electronic structure of undoped anatase TiO_2_ and carbon-, nitro­gen- and sulfur-doped TiO_2_. This semi-empirical methodology allowed for mapping the complete electronic structure of occupied (valence band) and unoccupied (conduction band) states of 3*d*-based materials. RXES is a photon-in photon-out technique, in which the intensities and energies of the incoming and emitted X-rays are monitored (Sá *et al.*, 2013 ▸; Baker *et al.*, 2017 ▸; Vankó *et al.*, 2006*a* ▸). In particular, by tuning the incident energy around the absorption edge of the element of interest, the unoccupied states are probed by electron excitation to the intermediate atomic state. Simultaneously, the information about the occupied electronic levels is obtained by the detection of specific X-ray photons emitted during the decay of the intermediate state to the final atomic state (the created core hole is filled by another core-level or valence-shell electron). Therefore, RXES combines X-ray absorption spectroscopy (XAS) and X-ray emission spectroscopy (XES) in one experiment, allowing for detailed investigation of the electronic structure of the matter, with element selectivity. Finally, we would like to emphasize the penetrating nature of the X-rays, which allows probing of the material structure with elemental selectivity in an ambient/working environment, *i.e.* experimental conditions that are not achievable for electron-based methods.

In this paper, we describe the application of a methodology for determining the electronic structure of metal-oxides with 5*d* transition metal elements. Among those, tungsten compounds represent an important class of materials due to their large variety of compositions, crystal structures and physical/chemical properties. They cover a wide range of electronic properties from metals to semiconductors and are therefore used in many technological applications. For the purpose of this work, metallic tungsten and tungsten (VI) oxide were selected. Cases of metal and metal-oxide materials are evaluated in order to show capability to simultaneously assess composition of around-Fermi electronic states, band gap energies and strengths of crystal field splitting. It is noteworthy that tungsten (VI) oxide (WO_3_) is regarded as an important multifunctional material with interesting chemical, electrical, optical and structural properties (Wang *et al.*, 2018*b* ▸; Zhu *et al.*, 2014 ▸). These are in turn determined by the electronic structure of a material (Wang *et al.*, 2011 ▸). Although there are a number of studies of 3*d* elements by means of high-resolution X-ray spectroscopies (see review article by Glatzel & Bergmann, 2005 ▸), 5*d*-based materials get far less attention (Kalinko *et al.*, 2020 ▸; Kotani *et al.*, 2012 ▸). With regards to tungsten, works of Yamazoe *et al.* (2008 ▸) and Smolentsev *et al.* (2011 ▸) are noteworthy. In particular, Yamazoe *et al.* (2008 ▸) analyzed the X-ray absorption fine-structure (XAFS) spectra of W *L*_3_-edges in order to determine the structure of various W species, *e.g.* WO_3_. The authors used the second derivative of the W *L*_3_-edge white line to calculate the splitting of 5*d* orbitals by the ligand field. A relatively low resolution of standard *L*-edge XAS spectra is caused by an intrinsic broadening from the core-hole lifetime. In the case of the 5*d* elements discussed, the 2*p* core hole in the excited state has a very short lifetime, broadening the bandwidth by about 4.81 eV (Campbell & Papp, 2001 ▸). In order to gain insight into the electronic structure of W oxides (WO_2_ and WO_3_), Smolentsev *et al.* (2011 ▸) employed RXES across the *L*_2_- and *L*_3_-edges. In combination with theoretical analysis, the authors showed that the differences between the *L*_2_- and *L*_3_-edges can be reproduced by taking into account spin-orbit interactions. While valence-to-core (v2c) XES spectra are extensively measured for 3*d* and 4*d* elements, the data for *L*-edge X-ray emission spectroscopy of 5*d* elements are very scarce (Kleymenov *et al.*, 2011 ▸; Glatzel *et al.*, 2010 ▸). As an example, Glatzel *et al.* (2010 ▸) used hard X-ray spectroscopy, in particular the v2c XES spectra around the *L*_3_ ionization threshold, to map the highest occupied electronic states of Pt nanoparticles under *in situ* conditions (adsorption of CO molecules). It has been shown that opening of the *d*-electronic band can be detected when a CO molecule is adsorbed on a Pt surface. The research demonstrated great potential of a combination of XAS/XES spectroscopies to map the around-Fermi electronic structure of 5*d* elements. In contrat to previous studies, we demonstrate the applicability of simultaneous measurement of high-resolution XAS and XES spectra to map the electronic structure of unoccupied and occupied states, respectively. In addition, this study shows the directions and strengths of changes in the electronic structure probed by hard X-ray spectroscopy methods when transitioning from metal to metal-oxide phases. Finally, we would like to emphasize that our studies were aimed at understanding electronic structure determined by a combination of X-ray spectroscopy methods to probe tungsten-based materials in a working chemical environment and with bulk sensitivity. Conventional electron-based techniques, such as X-ray photoelectron spectroscopy (XPS), are often unable to pinpoint the metal electronic states as they cannot be carried out under working conditions. Therefore, although there are numerous XPS studies on tungsten-based materials (Bittencourt *et al.*, 2005 ▸; Shpak *et al.*, 2007 ▸), direct quantitative comparison with X-ray spectroscopy measurements is not straightforward. The X-ray emission and absorption spectra represent initial and final electronic state distributions that are influenced by the effects of core-hole screening, natural lifetime broadenings of initial and final states, and transition probabilities (Glatzel & Bergmann, 2005 ▸). In contrast to the results given by XAS/XES methodology, XPS can address only the information about the occupied electronic state (final state). Moreover, the major limitation of electron-based techniques is they cannot be used for *in situ*/*operando* studies.

## 2. Experimental   

In this methodological development study, two commercial samples were investigated. A tungsten metal disc (from Goodfellow, 99.95%), 10 mm in diameter and 1.0 mm thick, was used without further purification. Nanopowder of tungsten (VI) oxide with a particle size below 100 nm (from Sigma–Aldrich) was pressed into pellets approximately 2 mm thick.

The experimental data were obtained at the SuperXAS beamline of the Swiss Light Source, located at Paul Scherrer Institute, Switzerland. The X-rays delivered from a super-cooled bending magnet were monochromated with a pair of Si(111) crystals. Downstream of the monochromator, the X-ray beam was focused with a toroidally bent mirror to a 100 µm × 100 µm spot size on the sample. The incident photon energy was calibrated to the tungsten metal *L*-edge energy, using the reference value of 10206 eV (*e.g.* Jayarathne *et al.*, 2014 ▸). The X-ray fluorescence from the sample was recorded using a wavelength-dispersive spectrometer in the von Hamos geometry (Szlachetko *et al.*, 2012 ▸). The sample studied was located at 45° to the incidence beam, whereas the X-ray spectrometer crystal was placed at 90° to the incidence beam. The experimental geometry was identical for both measured samples and the measurements were performed in an ambient environment (non-vacuum conditions). The collected spectra were not treated for self-absorption effects. It should be noted that samples were measured under the same experimental conditions and thus the relative spectral RXES/XAS features will remain unchanged. In the case of XES spectra, the incidence and emission X-ray energies are fixed and therefore the spectral shape is independent of self-absorption effects (effective sample thickness). The spectrometer was equipped with a cylindrically bent Si(111) crystal and operated in the fourth diffraction order at a Bragg angle of around 70.3°. The diffracted X-rays were recorded using a 2D Pilatus detector. For X-ray energies around the W *L*_3_-edge (∼10.2 keV), the experimental setup provides an energy resolution of about 1.5 eV. The RXES planes were collected by continuously scanning the incident X-ray energy around the W *L*_3_-edge (10208 eV) and recording the spectrum of the emitted X-ray photons (*L*α_1_ X-ray fluorescence line) from the sample. In addition, the non-resonant v2c XES spectra were measured at a fixed X-ray energy above the ionization threshold (*i.e.* using X-ray photons with 10 250 eV energy).

Theoretical calculations of high-resolution X-ray absorption spectra and v2c XES spectra were performed using real-space Green’s functions, as implemented in the program *FEFF9.6* (Rehr *et al.*, 2009 ▸, 2010 ▸). *FEFF9.6* is a self-consistent multiple-scattering code, able to simulate simultaneously the excitation spectra and the electronic structure of materials based on the structural information as input files. All calculations were performed using the Hedin–Lundkvist exchange potential and the final state rule for core-hole screening potential. *FEFF* calculations were performed for scattering radii of 5 Å, using atom distributions and arrangement from W (body-centered cubic) and WO_3_ (monoclinic) structures, which were taken from the *Crystallography Open Database* (http://www.crystallography.net/cod/search.html).

## 3. Results and discussion   

**Figure 1:**
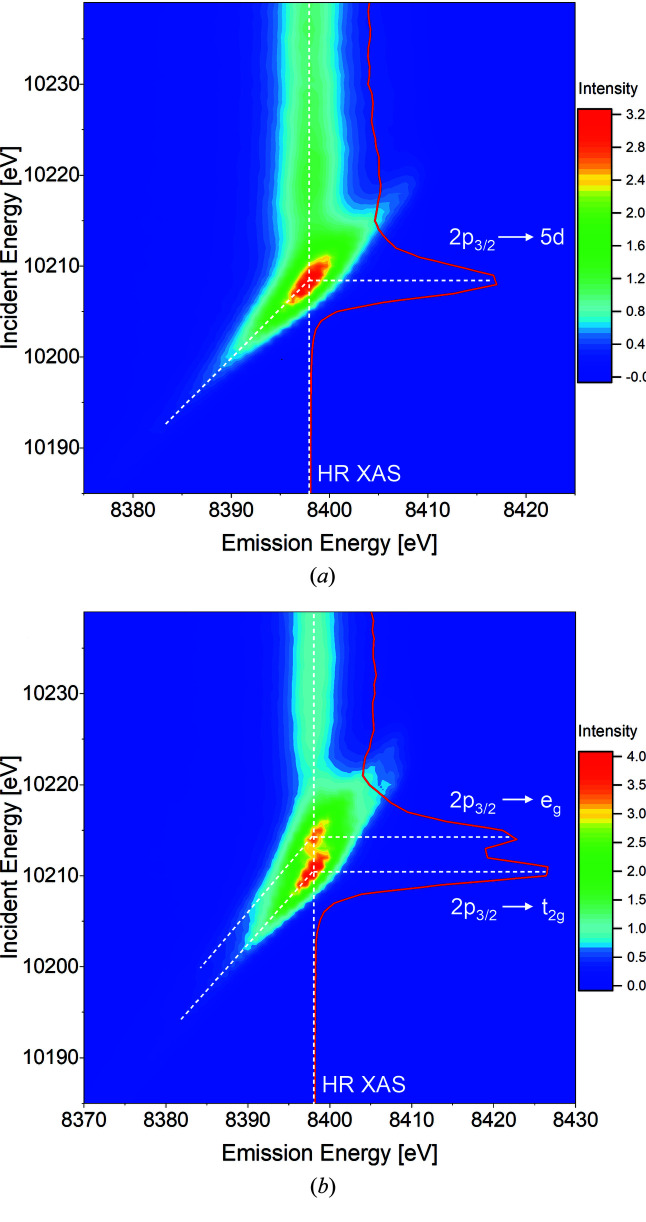
RXES planes measured around the W *L*_3_ absorption edge for (*a*) W and (*b*) WO_3_ materials. The diagonal dashed lines mark the 2*p*_3/2_ → 5*d* resonance; the vertical dashed lines illustrate the cuts along the planes to extract HR-XAS spectra. The extracted HR-XAS spectra are plotted as red lines on the planes.

The experimental RXES planes for metallic tungsten and tungsten (VI) oxide materials are presented in Figs. 1 ▸(*a*) and 1(*b*), respectively. The 2D maps were constructed by tuning the incident energy around the *L*_3_-edge and detecting the *L*α_1_ signal, which results from filling the created 2*p*_3/2_ core hole with 3*d*_5/2_ electrons (3*d*_5/2_ → 2*p*_3/2_ electron transition). It is important to emphasize that the application of dipole-allowed excitation (2*p*_3/2_ → 5*d*) and decay (3*d*_5/2_ → 2*p*_3/2_) electronic paths provides the strongest signals. For comparison purposes, the RXES planes were normalized, employing procedures typically used for XAS spectra. First, the 2D background function was determined for incidence energies below the ionization threshold and emission energies away from the main emission peak. The so-obtained 2D function of the background was then subtracted from the full RXES plane. Subsequently, for the highest incidence energies (*i.e.* 10240 eV) the integral of XES was normalized to 1. In this way, the projected XAS function from the RXES plane is normalized to 0 and 1 values at below and above edge energies, respectively. The dominant feature in the W RXES plane is strong 2*p*_3/2_ → 5*d* resonance with a maximum located at an incident energy of 10208 eV (diagonal dashed line). This resonance is present due to the partially unfilled *d* orbitals of metallic tungsten, which has a 5*d* ^4^ electron configuration. The strength and position of this resonance directly reflects the occupancy and chemical state of 5*d* elements (Cho *et al.*, 2012 ▸; Szlachetko *et al.*, 2014*b* ▸). The RXES plane for WO_3_ shows significant differences in terms of structure and intensity compared with the RXES plane acquired for metallic W. Due to the fact that WO_3_ has a distorted octahedral structure, two intense resonances are observed in the RXES plane with maxima at incident energies of 10 210 eV and 10 214 eV. Resonances appear due to splitting of the W 5*d* states into the W *t*_2g_ and *e*_g_ orbitals by the crystal field of the surrounding oxygen atoms. Previous reports suggest that an ideal octahedral structure would lead to a larger splitting of the 5*d* states (Jayarathne *et al.*, 2014 ▸; Yamazoe *et al.*, 2008 ▸). Detailed analysis shows that the main resonances have greater intensity and are shifted to higher incident energies compared with metallic W. This is caused by the higher oxidation state of W species in oxide form (+6) and therefore a higher number of unoccupied 5*d* states (*d* ^0^ configuration), which is in accordance with the energy conservation formula.

**Figure 2:**
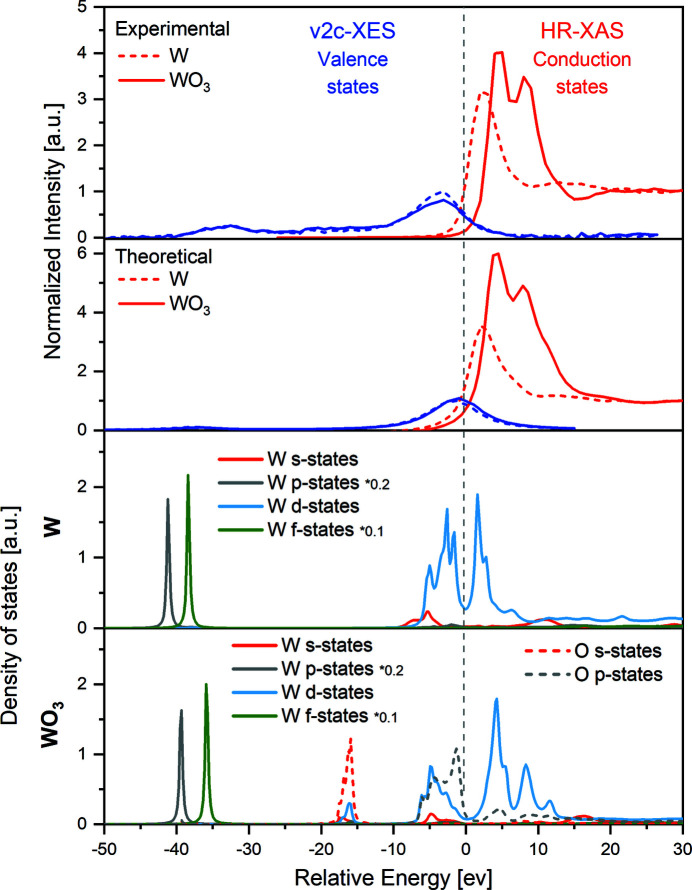
Top panels: comparison of experimental and calculated X-ray emission and X-ray absorption spectra for W and WO_3_ samples. Bottom panels: density of electronic states reflecting W and O orbital composition for metal and metal-oxide materials.

In order to gain insight into the electronic structure of the studied tungsten species, a combination of experimental data with theoretical calculations was used. The unoccupied electronic states (conduction band) were probed by high-resolution X-ray absorption (HR-XAS) spectra, obtained by cutting the RXES planes across the most intense emission energy. It should be mentioned that the *L*α_1,2_ signals were calibrated to reference W metal energies, assuming no XES chemical shift for the 3*d* → 2*p* transition. Thus, the corresponding HR-XAS curves for W and WO_3_ samples are extracted at the 8398 eV *L*α_1_ peak maximum. The occupied electronic states (valence band) were extracted from the non-resonant v2c X-ray emission (v2c-XES) spectrum, recorded at an incident energy above the ionization threshold, *i.e.* 10250 eV. The energy calibration of v2c-XES spectra was performed with elastically scattered photons by measuring the position of the elastic peak with the X-ray energy tuned around the v2c transition. The position of the elastic peak was determined by fitting the Gauss function to each spectrum. Finally, for intensity normalization, the v2c-XES spectra were normalized to the unit area for both studied samples following the method described by Vankó *et al.* (2006*b* ▸). It is worth mentioning that the non-resonant XES spectrum is much broader due to the width of the initial (2*p*_3/2_) and final states involved in the transitions. On the other hand, the HR-XAS spectrum (representing an intensity profile at maximum emission intensity) shows more detailed information due to the partial subtraction of initial state broadening that is seen as a diagonal dashed line (for 2*p*_3/2_ → 5*d* resonance) in the RXES plane (see Fig. 1 ▸). For both materials, the HR-XAS and v2c-XES spectra were scaled to the Fermi energy by subtracting a value of 10 206 eV. The latter was determined from the position of the inflection point of the high-energy side of the valence states (*L*_3_-edge ionization threshold) (Szlachetko & Sá, 2013 ▸). For data interpretation, the recalibrated and normalized HR-XAS and v2c-XES spectra are plotted together in Fig. 2 ▸ (top panel) in red and blue, respectively. Additionally, Fig. 2 ▸ presents a comparison of experimental spectra and theoretically simulated curves (top panels). As shown, the theoretical calculations are in good agreement with the measured XAS and XES profiles and therefore reproduce the measured spectral features well. We should note here that, in order to match the theoretical calculations with the experimental data, the simulated XES and XAS simulated profiles had to be shifted by the same energy value. The adjustment is necessary in order to account for an absolute energy mismatch given by the calculations.

The pronounced resonances observed on the RXES maps for both W and WO_3_ are visible in HR-XAS spectra as intense *L*_3_-edge white lines. The W-metal spectrum shows a single white-line peak, whereas the oxidized W species white line exhibits a distinct split due to the ligand field and a slight shift towards greater values. WO_3_ is composed of distorted octahedral WO_6_ units. According to crystal field theory, in octahedral splitting the *t*_2g_ orbitals appear at a lower energy than the *e*_g_ orbitals. From the relative energy separation of the W *t*_2g_ and *e*_g_ states, the crystal field splitting is found to be Δ_oct_ ≃ 3.7 eV, which is in good agreement with the previously reported values for WO_3_ (Triana *et al.*, 2017 ▸; Yamazoe *et al.*, 2008 ▸). However, we would like to stress that the RXES measurements allow for direct crystal field splitting characterization unlike conventional XAS methods (Triana *et al.*, 2017 ▸; Yamazoe *et al.*, 2008 ▸), which use second-derivative procedures on the X-ray absorption curves. In this way, the RXES methodology and the resulting high-energy resolution XAS functions will allow for determination of much smaller splitting energies. Due to the fact that crystal field splitting arises from the interaction of ligands with metal orbitals, the magnitude of the splitting depends on the nature of the metal ion and the ligand as well as can be ascribed to the local structural disorder in the first and second coordination shells. From the valence-band site, the collected v2c-XES spectra suggest negligible changes in the density of electronic states. However, it should be noted that the intensity of valence transitions is proportional not only to the number of electrons in orbitals, but also to the transition probability and level of hybridization of electronic states.

The experimental spectral features can be interpreted based on the density of states calculated with the *FEFF9.6* code. The particular orbital contributions, namely *s*, *p*, *d* and *f* states of tungsten and the *s* and *p* states of oxygen, are plotted in Fig. 2 ▸ (bottom panels). For metallic W the unoccupied and occupied electronic states around the Fermi level consist mainly of W *d* orbitals, with negligible contribution from the *s* and *f* states. An additional feature can be observed at *ca* −35 eV, which is associated with the W *f* orbitals. The quadrupole transitions from the 4*f* orbitals are visible due to the strong overlap of the 2*p* and 4*f* wavefunctions (Smolentsev *et al.*, 2011 ▸). Although the W *p* states are shown in Fig. 2 ▸ for comparison with other orbitals, we would like to emphasize that its contribution to XES and XAS signals is expected to be small due to dipole selection rules for the excitation/transition processes and eventually is modulated by the strength of hybridization of *p* and other electronic orbitals. With regards to WO_3_, the main contribution to the conduction band comes from the un­occupied W *d* states. Additionally, a minor contribution from O *p* orbitals is noticeable. Theoretical calculations show also that unoccupied *d* orbitals are split due to the ligand field and shifted to higher energy compared with the W case, which is in good agreement with experimental data. In the occupied electronic states of WO_3_, three main structures may be distinguished. The v2c peak lying just below the Fermi energy (−3.5 eV) can be assigned to W *d* orbitals and O *p* orbitals. This observation suggests that there is a considerable amount of hybridization between these states, because they partially overlap in the energy scale. Nevertheless, in contrast to metallic W, the upper level of the valence band is mainly composed of O 2*p* states. Moreover, an additional small structure is observed at *ca* 16 eV, which is related to the presence of O *s* orbitals mixed with the small contribution from W *d* states. No significant changes are observed for the peak located at *ca* −35 eV, which is assigned to the W *f* orbitals. We should note that the calculated density of states, based on Green’s function theory, are consistent with the ones reported by Smolentsev *et al.* (2011 ▸) and Ping *et al.* (2012 ▸) which used time-consuming density functional theory methods to determine the WO_3_ band structure.

As shown, the measured v2c-XES and HR-XAS spectra are well reproduced by the theoretical DOS calculations and thus provide detailed information about the electronic states of W and WO_3_. The W state is characterized by a gapless unoccupied and occupied electronic structure (the estimated band-gap-like energy is equal to *ca* 0.2 eV), which is consistent with the results expected for the metallic phase. On the other hand, the WO_3_ shows semiconducting behaviour, *i.e.* it exhibits an energy band gap of ∼3.0 eV between the measured occupied and unoccupied electronic states. The band-gap energy as well as the scaling of the energy to the Fermi level were derived following the procedure described by Chiou *et al.* (2007 ▸) and Preston *et al.* (2008 ▸) as well as in our previous works (Szlachetko & Sá, 2013 ▸). Chiou *et al.* (2007 ▸) obtained the band gap value by extrapolating the leading edges in the XANES and XES spectra (O *K*-edge XANES and O *K*α XES spectra) to the baselines, which correspond to the conduction-band minimum (CBM) and valence-band maximum (VBM), respectively. In our data, due to the relatively large final state broadening in XES spectra, the band-gap like energy was extracted from infection points of v2c-XES and HR-XAS curves. Interestingly, as proved by our results, the transition from metallic state to metal-oxide is coupled with a significant shift of unoccupied/conduction states, while occupied/valence states show very small changes. The W and WO_3_ 4*f* electronic states remain unchanged while a positive XES signal change at −18 eV and a negative signal change at −4 eV are observed at positions corresponding to the W 5*d* orbital mixed with O *s* and O *p* states, respectively. It is also important to note that the energy positions of the highest occupied states for W and WO_3_ remain virtually unchanged. The results suggest that the contribution of the valence state, as probed by hard X-ray spectroscopy, will have less impact on the structure of the total electronic states compared with the distribution of unoccupied electronic states when forming metal-oxide material. As a consequence, modifications via the doping method have to be considered to shift the valence potential for efficient solar-driven photocatalytic reactions, especially water splitting. Finally, we would like to discuss band gap-like information that may be extracted from non-resonant and resonant XES, which indeed may differ in absolute value from the values obtained by optical spectroscopy methods. This is mainly because the estimated energy band gap value is affected by core-hole screening effects (Szlachetko *et al.*, 2014*a* ▸). In other words, during the scattering process a deep-lying core-hole interacts with outer-shell electronic states before a final transition from the 3*d* to 2*p* level occurs. It is noteworthy that the measured positions of valence and conduction states are affected not only by the final hole state but also by changes in the hybridization of electronic states and excitation probabilities due to the contribution of additional orbitals. This implies that the described methodology gives generic band-gap-like information and the composition of v2c-XES and HR-XAS spectra provides rather a picture of projected electronic orbitals than precise information on the band gap energy. Thus, the estimated energy band gap value should be regarded as an averaged distance between the *d* projected occupied and unoccupied density of states. Nevertheless, associated changes in the relative positions of the energy band edges provide valuable information regarding electronic structure and depend on the elemental composition as well as the presence of defects or lattice distortions.<!?tpb=12pt>

## 4. Conclusions   

In summary, we have shown that the combination of resonant and non-resonant X-ray emission spectroscopies and theoretical modelling can provide direct quantitative analysis of 5*d* metal-oxide total electronic structures (occupied and unoccupied states). Metal and metal-oxide material cases studies (W and WO_3_, respectively) were evaluated to show the ability of the approach to assess simultaneously the composition of the electronic states around-Fermi, band gap energies and strength of crystal field splitting. Composition of occupied and unoccupied electronic states of a material in the vicinity of Fermi energies is vital because it underpins its physical, chemical and mechanical properties. The results obtained in the present study are important for the rational design of metal-oxide materials and may be adopted to study other transition metal compounds with different composition, morphology and structures. Additionally, the approach can be adapted to any crystallite size, even ones where the diffraction signal is limited or non-existent.
